# Ceria-based coatings on magnesium alloys for biomedical applications: a literature review

**DOI:** 10.1039/d2ra06312c

**Published:** 2023-01-09

**Authors:** V. Hernández-Montes, R. Buitrago-Sierra, Mónica Echeverry-Rendón, J. F. Santa-Marín

**Affiliations:** a Universidad Nacional de Colombia. Sede Medellín. Facultad de Minas. Medellín, Colombia, Grupo de Tribología y Superficies Medellín Colombia vanehdezm@gmail.com; b Instituto Tecnológico Metropolitano (ITM). Facultad de Ingenierías, Grupo de Materiales Avanzados y Energía (MATyER) Medellín Colombia; c IMDEA Materials Institute Calle Eric Kandel 2, 28906 Getafe Madrid Spain

## Abstract

Magnesium alloys are being studied for use in temporary orthopedic implants because of their mechanical properties, which are similar to those of human bone, and their good biocompatibility. However, their application is limited due to their rapid degradation, and early loss of their mechanical properties, decreasing the stability of the implant and its proper synchronization with tissue regeneration. In this regard, various surface coatings have been used to improve their biological, physico-chemical and biodegradation properties. Currently, one of the most explored strategies is using smart coatings because of their self-healing properties that can slow down the corrosion process of Mg and its alloys. Ceria-based materials show promise as coatings for these alloys. Their unique redox capacity not only provides Mg alloys with good self-healing properties but also interesting biological properties, which are described in this paper. Despite this, some problems and challenges related to the biocompatibility and application of these materials in coatings remain unsolved. In this article, a critical review is presented summarizing the most representative literature on ceria-based coatings on Mg alloys for their potential use as biomaterials. The results show that ceria is a versatile material that may be used in industrial and biomedical applications.

## Abstract 2

Current research trends in Mg alloys are focused on the development of smart coatings with self-healing properties that improve corrosion resistance of these alloys. Ceria based materials have recently emerged as materials that can be used as smart coatings on Mg alloys.

## Graphical abstract

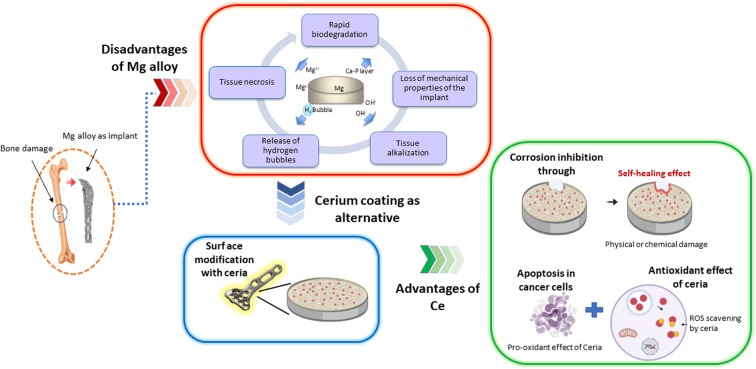

## 1. Introduction

Bone tissue diseases significantly reduce patients' quality of life because some of their medical signs and symptoms include acute pain, loss of function, bone segment deformation, hematomas, and abnormal mobility, which can lead to long-term physical disabilities. In addition, bone trauma by different causes is highly prevalent today. As indicated by Cordero *et al.*,^1^ it is estimated that between 779 to 1574 per 100 000 people from low- and middle-income countries experience musculoskeletal injuries every year. Osseous tissue has an intrinsic ability to regenerate and repair itself through a bone remodeling process associated with new bone formation and resorption,^2^ allowing for the spontaneous healing of small bone defects^3,4^. However, this regeneration process is slow, and its success is subject to the age of the patient and the degree of tissue loss.^5^

Bone implants are required to repair fractures by realigning and fixing the injured bone to promote proper healing.^6^ Nevertheless, commercial metal implants for fracture fixation generate substantial costs to the healthcare industry (approximately USD 708.37 per clinical patient)^7^ because a second surgical procedure is necessary to remove the temporary implants. This second surgery typically has implications for the patient, including new fractures after the material is removed, increased undesirable volume in the site. In addition, aesthetic issues are also related to new scars at the explantation site. It also requires additional hospital stays and time away from work (an average of two days for the former and 15.4 days for the latter).^7^ As an alternative option for this situation, biodegradable medical implants are under research to bring a new therapeutic option with better results in terms of medical care cost, decreasing the pain and discomfort of the patient and offering a structural platform for tissue regeneration.^4^

In recent years, biodegradable magnesium (Mg) alloys have been one of the most studied materials for bone implant applications.^8–10^ However, Mg alloys are highly susceptible to degradation under a physiological environment and therefore lose their mechanical strength prematurely, before the time necessary for the bone implants to remain stable (approximately 12 weeks).^11^ Consequently, their degradation should be controlled at a reasonable rate.^12^ The use of dense and stable coatings has been proposed as a physical barrier to slow down the corrosion process^13^ and improves the direct structural and functional connection between the implant and the bone (osseointegration). To date, different nature of materials has been used for coating such as hydroxyapatite, titanium dioxide, silane, magnesium oxide, *etc.* However, these coatings are susceptible to damage because of corrosive environments. Thus, the design of smart coatings with novel materials capable to respond to various stimuli should be explored.^13^

Some smart coatings have self-healing properties, which provide long-term corrosion protection to the substrate.^14^ Examples of smart self-healing coatings applied to Mg alloys include encapsulation coatings, layer-by-layer self-assembly coatings (LBL) and chemical (organic/inorganic) conversion coatings.^13^ Some of these coatings include the encapsulation of corrosion inhibitors as healing agents (ions or nanoparticles), which provide the coating with barrier and active properties through internal or external stimuli such as ion release.^13^ Active repair agents such as 8-hydroxyquinoline (8-HQ), benzotriazole (BTA), and cerium ions, are incorporated directly into the host coating on Mg alloys.^15^ In the event of local corrosion, they react with the hydroxyl ions and magnesium hydroxides to form an insoluble precipitate, thus allowing the defects to be repaired.^15^

Cerium oxide nanoparticles (NPs) are inorganic materials with the potential for usage as smart self-healing protective coatings.^16^ Cerium oxide (CeO_2_), also known as ceria, is a rare earth metal oxide that can switch its oxidation states between Ce^3+^ and Ce^4+^,^17^ allowing for the formation of “reactive sites'’ in its structure.^18^ It can adopt a fluorite-like cubic structure since it has a highly reactive surface for neutralizing free radicals.^19^ According to a recent review article by Inbaraj and Chen,^20^ CeO_2_ NPs have applications in biomedical sciences because they have anti-inflammatory and antioxidant properties and the ability to stimulate the migration and proliferation of keratinocytes, fibroblasts, and vascular endothelial cells. The use of CeO_2_ NPs in biological systems has sparked a lot of interest due to their redox cycling potential and ability to switch between oxidation states.^21^

However, because the cytotoxicity of CeO_2_ NPs has not been completely determined, there is much controversy about their use in biomedical applications. Despite this, several recent publications have highlighted their antioxidant and pro-oxidant properties and their important role in different biological responses. Therefore, the aim of this paper is to present a review of ceria-based coatings deposited on Mg alloys to improve their corrosion resistance, as well as to address the controversial issues surrounding their biocompatibility.

## 2. Magnesium-based biomaterials

Magnesium (Mg) is the second most abundant intracellular cation present in the human body, and it plays a fundamental role in different organs.^22,23^ Recommended daily allowance for Mg is 240–420 mg per day. However, the body can eliminate Mg breakdown products easily and the products are tolerated by the host without immunogenic or mutagenic tendency.^23^ The body can tolerate an excess of Mg ions, since they can be transported through the circulatory system and excreted through urine and feces, without causing adverse effects.^24^ Hence, it is not only a biodegradable but also a bioabsorbable material.^25^ Furthermore, *in vivo* tests have shown that Mg^2+^ cations released from Mg-based biomaterials during the corrosion process can promote bone regeneration and speed up the healing process.^26,27^

Despite all this, the degradation of Mg is very fast in an aqueous solution, for example, Abdel-Gawad and Shoeib reported that pure magnesium has a corrosion rate of about 5.01 millimeters per year in simulated body fluid (SBF).^28^ During this process, hydrogen evolution occurs, representing risk in the biocompatibility by forming gas pockets between the material and tissue.^29^ On the other side, the presence of magnesium hydroxide, as a corrosion product, increases the pH of the surrounding environment. Both events, hydrogen evolution and alkalinization of the area, may alter the homeostasis of the treated area and in some cases cause necrosis of the tissue. Therefore, the rapid corrosion of these materials inside the human body remains a major impediment to their clinical application.^30^ Despite, controlled degradation of Mg alloys could prevent issues such as implant failure due to loss of the mechanical integrity, and intolerable hydrogen evolution.

Due to the low corrosion resistance of pure Mg, two main strategies are under investigation: the manufacture of new alloys and obtaining surface coatings. With respect to alloys, several families of alloys such as: Mg–Al, Mg–Ca, Mg–Sr, Mg–Zn, Mg–Si, Mg–Sn, Mg–Mn, Mg–Re, and Mg–Ag, have been developed to enhance its properties and promote its use as biomedical implants. Biocompatibility of Mg alloys, however, depends on their components. Current studies have focused on synthesizing and characterizing Mg-based biomaterials with a variety of compositions to control their properties. The selection of a proper alloy that fulfil the requirements of biocompatibility are essential to keep the biocompatibility of the implant.

Further, the development of coatings with active materials that provide better corrosion resistance for Mg alloys is one of the main focuses of current research. CeO_2_ particles are a suitable option because they are corrosion inhibitors and provide self-healing capabilities.^31^ Both properties are obtained when CeO_2_ is added to a coating, as stable cerium hydroxides are formed by interaction with the OH^−^ ions released during the corrosion process. Furthermore, the human body has a highly corrosive environment for Mg alloys. The presence of ions in the physiological medium such as chloride (Cl^−^), causes damage to the surface of Mg alloys and increases the corrosion rate. However, it has been reported that Cl^−^ ions can be incorporated into surface oxygen vacancies of CeO_2_, and this reaction has proven to be quite stable. Accordingly, less free Cl^−^ ions are available in the medium and the degradation process is delayed.^32^ Calado *et al.*^32^ and Anjum *et al.*^33^ demonstrated that coatings on Mg alloys containing ceria NPs can create a barrier against corrosive ions, stabilize corrosion products, and block the electrolyte diffusion pathways within the coating.

## 3. Biocompatibility of cerium oxide: the controversy

Ceria-based coatings have proven to have redox properties.^34^ Various routes for the synthesis and design of ceria-based materials have been thoroughly studied in recent decades because they directly influence their biological properties.^35^ The production of ceria-based materials with different morphologies (cubic, spheres, rodlike and polyhedral) and sizes (nanometric and micrometric) has been reported, and the evaluation of CeO_2_ NPs is currently a hot topic for researchers.^36^ However, the biocompatibility of these NPs has not been well determined. There is a strong correlation between the cellular effects of these NPs and their properties, including synthesis, morphology, particle aggregation, and surface charge.^37^

Table 1 shows the different biocompatibility results for CeO_2_ NPs reported in the literature. As observed in this table, there is great variability in the concentration of CeO_2_ particles evaluated by the different authors, which range from 0.0005 to 200 mg ml^−1^. Commercial CeO_2_ particles, as well as synthesis methods like wet chemistry and green methods, have been used. Cytotoxicity, genotoxicity, and oxidative stress assays are commonly employed to evaluate their biological response *in vitro* in cancerous and normal cell lines such as human IMR-32 neuroblastoma and human periodontal fibroblast cells, respectively. In addition, *in vivo* tests have been performed routinely in animal models such as mice and rats. Implantation tests have also been conducted in specific organs to assess the histopathological effects, immunotoxicity, cytotoxicity, and genotoxicity of cerium particles. However, their effect in cell lines or animal models has not been well determined, as the results of different studies show controversial conclusions. These particles have been shown to cause cytotoxicity and genotoxicity in some studies, while no systemic toxicity has been reported in others. For instance, L. Alili *et al.*^38^ found that CeO_2_ NPs exhibited selective cytotoxic effects on human melanoma cells and no cytotoxic effects on normal (stromal) cells. They reported that the cytotoxic effect of these NPs depends on the redox-active function of the material and that they possess great selectivity towards cancer cells. According to their results, the viability of normal cells was not altered at 96 h after treatment with CeO_2_ NPs, while melanoma cells showed a decrease in viability of around 45%.

**Table 1:** Biocompatibility studies of ceria particles

Type of particle/synthesis method	Size	Concentration	Evaluation test	Cell line or animal model	Effect	Ref.
***In vitro***						
Commercial NPs (CAS no. 1306-38-3) and microparticles (MPs) (CAS no. 1306-38-3) from Sigma-Aldrich	CeO_2_ NPs: <25 nm, CeO_2_ MPs: <5 μm	0.0125, 0.025, 0.05, and 0.1 mg ml^−1^	Allium and comet tests	Allium cepa root meristematic cells	MPs and NPs showed cytotoxic and genotoxic effects	48
Commercial NPs (CAS no. 1306-38-3) and MPs (CAS no. 1306-38-3) from sigma chemical co., Ltd.	CeO_2_ NPs: <25 nm, CeO_2_ MPs: <5 μm	10, 20, 50, 100, and 200 mg ml^−1^	Genotoxicity, cytotoxicity, and oxidative stress assays	Human neuroblastoma cell line (IMR32)	CeO_2_ NPs caused size- and dose-dependent toxicity. CeO_2_ MPs did not generate any significant changes in the cells	49
Green method using fresh egg white	CeO_2_ NPs: 24.2 nm	0.0125, 0.025, 0.05, 0.1, 0.2, 0.4 and 0.8 mg ml^−1^	Cell viability by MTT assay	Human periodontal fibroblast cells	No toxicity was observed even when high doses were tested	50
***In vivo***						
Not reported	CeO_2_ NPs: 4–8 nm	0.0044, 0.0088, 0.0176, and 0.0352 mg per aspiration	Immunotoxicity, histopathological studies and biodistribution of NPs	C57Bl/6 mice	CeO_2_ NPs caused non-dose-dependent DNA damage and inflammation. No systemic genotoxicity was observed	51
Commercial NPs from strem chemicals, Inc.	Not reported	250, 500, and 1000 mg per implantation site	Implantation study of NPs, systemic toxicity, and genotoxicity studies	Female wistar rats	CeO_2_ NPs showed no systemic toxicity or micronucleus induction in bone marrow. Local tissue reactions were minimal	52
Wet chemistry method	CeO_2_ NPs: 3–5 nm	0.0005 and 0.0025 mg ml^−1^	Liver implantation	CD-1 mice	Nanoceria administration showed no overt toxicity	53

CeO_2_ NPs are considered cytotoxic agents capable of killing cancer cells and have been labeled as pro-oxidant and pro-apoptotic agents.^39^ Their antioxidant activity has also been demonstrated by their protective effect against oxidant-induced apoptosis.^17,40^ For example, Pesic *et al.*^41^ studied their antioxidant activity in cell cultures, and although they found no adverse effects in two normal cell lines (NCL): MRC-5 and HaCaT cells lines, a moderate degree of cytotoxicity was observed in four cancer cell lines (CLs): DLD1, DLD1-TxR, NCI-H460 and NCI-H460/R cells line. These results indicate an alteration in the redox balance in CLs. Based on these differential responses between NCL and CLs, the selective killing of cancer cells was proposed by these authors. Similarly, Abbas *et al.*^42^ reported that CeO_2_ NPs were toxic to neuroblastoma cancer cells and non-toxic to healthy HEK-293 cells. Therefore, their ability to have a differential effect on NCL *versus* CLs has been recognized. This effect has been mainly attributed to the increased acidification of the cancer microenvironment and is associated with a response that is dependent on the level of intracellular Reactive Oxygen Species (ROS).^38,40^

CeO_2_ NPs are well known to exhibit vigorous antioxidant activity and have been reported to have catalase (CAT), superoxide dismutase (SOD), oxidase (OXD), peroxidase (POD) and phosphatase (AKP) mimetic activity due to their ability to switch between the Ce^3+^ and Ce^4+^ oxidation states depending on the environment.^43^ This unique redox potential can prevent cell damage caused by various ROS. Antioxidant activity to eliminate ROS is an enzyme-like activity that occurs when CeO_2_ NPs enter a normal cell *via* endocytosis and eliminate ROS such as peroxides, superoxides, and hydroxyl radicals due to (1) SOD-like activity, in which O^2−^ is reduced to H_2_O_2_ by oxidizing Ce^3+^ to Ce^4+^, and (2) CAT-like activity, in which Ce^4+^ is reduced back to Ce^3+^ by oxidizing H_2_O_2_ to molecular oxygen and water, thus protecting the normal cell.^18^

Various authors have examined the antioxidant activity of CeO_2_ NPs in different types of cells. For example, Fillipi *et al.*^44^ evaluated their ability to scavenge hydroxyl radicals in two different fluids. They found that these NPs exerted a high OH^−^ removal activity in phosphate buffered saline (PBS) and surrogate lung fluid (SLF). The OH^−^ removal efficiency of the NPs in the SLF reflects the redox activity of CeO_2_ under more realistic conditions.

Another study that demonstrates the antioxidant properties of CeO_2_ NPs was conducted by C. von Montfort *et al.*^45^ These authors found that CeO_2_ NPs reduced the viability of CLs (SCL-1: cutaneous squamous cell carcinoma) and had no cytotoxic effect on NCL (HDF: Human Dermal Fibroblasts). Also, their results suggest that these NPs, due to their good antioxidant properties, prevent cell death induced by ROS and promote cell proliferation. Likewise, S. Kim *et al.*^46^ synthesized Levan-coated Cerium Oxide Nanoparticles (LCNPs) with improved antioxidant activity. According to the results of the biological evaluations, modified CeO_2_ NPs reduced ROS levels when exposed to fibroblast cells (NIH3T3) that had been previously stimulated with hydrogen peroxide. LCNPs were found to have high antioxidant activity in NCL in a neutral physiological environment. In summary, the studies that have evaluated the antioxidant effect of CeO_2_ NPs in normal cell lines have reported a significant reduction in ROS production, as well as evidence of protection against oxidative stress. Although the findings indicate that CeO_2_ NPs have a neuroprotective and antioxidant role, more studies are needed to determine their clinical importance.

CeO_2_ NPs have also been shown to exhibit anticancer activity.^47^ They protect healthy cells from ROS but kill cancer cells by stimulating ROS production. Their anticancer activity is attributed to their pro-oxidant effect on cancer cells. CeO_2_ NPs can be taken up into cells by receptor-mediated endocytosis and then released into the cytoplasm through vesicle trafficking pathways. Notwithstanding, CeO_2_ NPs inhibit CAT-like activity, causing a large amount of H_2_O_2_ to accumulate in cancer cells. ROS also harm some cell organelles and cause dsDNA to be separated into single strands (DNA denaturation), resulting in apoptosis.^18^

Several authors have examined the pro-oxidant or anticancer activity of CeO_2_ NPs against different cancer cell lines. For instance, Datta *et al.*^54^ investigated the pro-oxidant activity of CeO_2_ NPs in HCT 116 (human colorectal carcinoma cell line) and demonstrated that treatment with CeO_2_ induces DNA fragmentation by increasing ROS production. This treatment causes cell death through p53-dependent apoptosis pathways. For their part, Sridharan *et al.*^55^ analyzed biosynthesized CeO_2_ NPs to determine their anticancer action in human breast cancer cells line (MCF-7). According to the authors, these NPs preferentially attacked MCF7 instead of normal cells.

Also, Z. Rasouli *et al.*^56^ synthesized and characterized CeO_2_ NPs with different morphologies with and without doping (CeO_2_ NPs, CeO_2_ NPs doped with nickel, hollow spherical CeO_2_ NPs, and hollow CeO_2_/SiO_2_ core–shell compounds). The cytotoxic effect of these materials on cancerous cells (HT-29: human colorectal adenocarcinoma cell line) and fetal normal cells (HFFF2: human Caucasian fetal foreskin fibroblast cell line) was evaluated using the MTT assay. The hollow CeO_2_/SiO_2_ core–shell compounds showed the highest anticancer effect on HT-29 when compared to the other materials. In addition, the cytotoxic effect of all compounds was found to be significantly lower on normal cells (HFFF2) than on tumor cells (HT-29).

E. Nourmohammadi *et al.*^47^ synthesized CeO_2_ NPs using the coprecipitation method and assessed their anticancer effects on the murine fibrosarcoma cell line (WEHI164). The results were then compared with those obtained in normal cells (L929). The cell viability (by MTT), apoptosis, and DC-FDA assays revealed that the NPs increased ROS levels and induced apoptosis in cancer cells (WEHI164) in a dose-dependent manner. Normal cells (L929) showed low levels of toxicity even at concentrations higher than 250 μg ml^−1^ in the MTT assay.

To summarize, several authors have evaluated the *in vivo* cytotoxic effects of CeO_2_ NPs on different cancer cell lines. According to the reported results, ceria could be used to treat cancer in the future. Ceria based materials have also been studied in recent years for bone applications.^39^ For example, J. Li *et al.*^57^ reported that CeO_2_ NPs show favorable biological responses and that, thanks to their mixed valence state, they could potentially regenerate bone tissue without using exogenous osteogenic inducers. Furthermore, ceria-based materials have been shown to significantly promote several biological processes such as the growth, migration, and osteogenic differentiation of human mesenchymal stem cells line.^58^ For their part, J. Xiang *et al.*^59^ found that CeO_2_ NPs improves the vascularization of bone grafts. Furthermore, bone-targeted pH-sensitive CeO_2_ NPs could provide a novel anabolic strategy for the treatment of bone disorders with excessive bone resorption.^60^

In conclusion, the unique redox potential of CeO_2_ NPs can protect the cells from damage caused by several ROS and kill cancer cells by inducing ROS formation. In fact, most studies have reported cell damage caused by these particles on cancer cell lines, but no damage has been observed in healthy tissue cell lines.

## 4. Smart self-healing coatings on magnesium-based biomaterials

Recent developments in Mg-based biodegradable materials for orthopedic applications have focused on improving corrosion resistance and biocompatibility.^61^ The purpose is to provide mechanical stability to the material, favoring its initial fixation and subsequent degradation. They are also expected to aid in the healing processes without causing any negative biological response.

Surface modification methods have been used to control the degradation rate of Mg alloy.^62^ Various organic and inorganic materials have been synthesized and evaluated for use as coatings on Mg alloys mainly in bone and cardiovascular applications.^63^ For instance, surface modification of Mg alloys using physical barrier coatings has been investigated. According to a review paper by D. Zhang *et al.*,^13^ metal-based coatings (including metal hydroxide and metal oxide coatings), polymer coatings, silane sol–gel coatings, calcium phosphate coatings, among other types of coatings, are commonly employed to protect Mg and its alloys.^13,63^

**Fig. 1:**
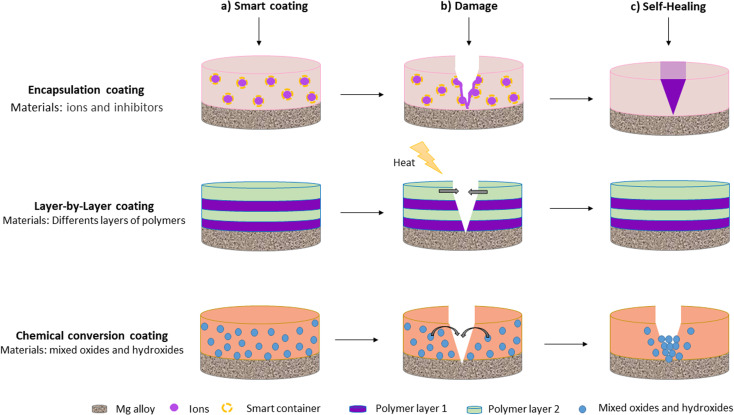
Schematic representation of self-healing coatings deposited on Mg alloys.

Nonetheless, when barrier coatings are immersed in complex environments such as physiological fluids, they suffer from early damage, and corrosion is accelerated. Therefore, recent studies have focused on the development of smart coatings with self-healing properties.^33^ Smart self-healing coatings are defined as those that can repair their physical damage and recover functional performance without the need for external intervention.^15,64^ Self-healing mechanisms usually restore the physical barriers of the coating by sealing or closing defects or by slowing down corrosion reactions at coating defects. Smart materials with self-healing properties are thus expected to significantly improve the corrosion resistance and service life of coatings deposited on Mg alloys.^15^ Self-healing coatings are typically obtained by chemical conversion, encapsulation, and layer-by-layer assembly. Fig. 1 provides a schematic representation of self-healing coatings deposited on Mg alloys.

Different materials such as CeO_2_ NPs, 8-hydroxyquinoline, mercaptobenzothiazole, and benzotriazole are used to produce self-healing coatings, with polymerizable healing agents and corrosion inhibitors being particularly important to achieve autonomous healing.^15^

Autonomous healing systems can repair the functional properties of the material surface without the intervention of external sources. There are other dependent methods employed to create a self-healing effect. These methods require the application of external stimuli such as heat or light.^65–67^ The action of these stimuli helps to self-heal the coating through the repair of intrinsic chemical bonds or physical conformations of the structure of a three-dimensional polymer network. Some of the materials typically used in such coatings are polypyrrole, polycaprolactone, and shape memory polymers^68^

P. Xiong *et al.*^69^ stated that a typical coating with self-healing properties consists of a host coating that acts as a physical barrier and a carrier for the corrosion inhibitor. Once the corrosive medium damages the barrier coating, corrosion inhibitors are activated and migrate to the defect sites forming a passive film that slows the progression of the corrosion process. The host is a substrate composed primarily of polymers with inhibitor loading capabilities. Micro-Arc Oxidation (MAO) coatings and Layered Double Hydroxide (LDH) coatings are two other types of host coatings.^13^

Corrosion inhibitors are often ions (such as Ce^2+^) or small molecules (such as 8-hydroxyquinoline and benzotriazole).^70^ They can form insoluble compounds in the corrosive medium during the degradation of Mg alloys. According to M. J. Anjum *et al.*,^33^ the self-healing mechanisms of Mg can be divided into three groups. In the first mechanism, the healing agent is incorporated into the formed coating layer (mainly oxide), and it heals the coating through a chemical reaction. In the second mechanism, healing agents form complexes or a chelate with the metal ions produced due to corrosive action and then deposit on the defective area to heal the coating. In the third mechanism, the coating has the ability to heal itself by forming an oxide layer or repairing bonds.^13,71,72^

Characterization techniques used to understand the self-healing mechanism of these materials include: (1). surface analysis: can be performed with optical microscopy, scanning electron microscopy and confocal laser scanning microscopy. It is generally used to evaluate the self-healing performance of coatings.^73–75^ (2). Analysis of chemical or elemental composition: includes analysis with Energy Dispersive X-ray Spectroscopy (EDS/EDX), X-ray Photoelectron Spectroscopy (XPS) and electron microprobe analysis (EPMA). These analyzes allow us to know the elements present in the damaged area and their oxidation state. Also, EPMA provide information on the migration of ions in the coating defect area. (3). Electrochemical test: electrochemical impedance spectroscopy (EIS), potentiodynamic polarization (PP) vibrating electrode scanning technique (SVET), scanning electrochemical microscopy (SECM) and selective ion electrode scanning technique (SIET) are also used to investigate the process of self-healing of coatings.^76,77^ Together, these techniques provide insight into the degradation rate of coatings, estimate corrosion kinetics, map local pH changes, and record and quantify the local electrochemical activity of coatings in real time. Which allows the observation of the action of healing agents or corrosion inhibitors on self-healing coatings.^73^

In general, CeO_2_ NPs have been recognized as materials with corrosion inhibition properties. Consequently, they have emerged as smart materials for self-healing coatings on Mg alloys and have great potential for biomedical applications.

### 4.1 Ceria based coatings on magnesium alloys

Ceria has been extensively studied in biomedical applications due to its ability to inhibit and mitigate the corrosion of metals and alloys, including Mg alloys.^78,79^ Cerium cations exhibit corrosion resistant and self-healing effects when incorporated in various coating formulations (CeO_2_ and others). Corrosion is inhibited by the interaction of ceria with OH^−^ ions released during the corrosion process and the formation of stable cerium hydroxides.^32^

**Fig. 2:**
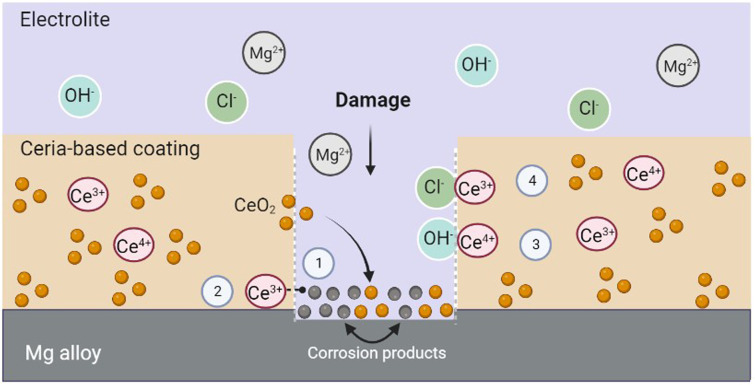
Possible self-healing mechanisms of cerium-based coatings. 1. The particles generate a physical blockage of the electrolyte diffusion pathways. 2. Ceria can stabilize the corrosion products of Mg alloys. 3. OH ions can adsorb on the ceria surface, and deposit on the damaged surface. 4. Ceria can adsorb chloride ions.

The self-healing mechanism induced by ceria is initiated after a defect is formed in the coating. Ceria's corrosion inhibition activity is thus primarily triggered by an increase in the concentration of hydroxyl groups at the corrosion sites.^14^ When the corrosion process is activated, cerium ions diffuse into the affected sites and precipitate as hydroxide and oxides.^80^ Furthermore, given ceria's tendency to adsorb water and/or hydroxyl ions, species such as magnesium hydroxide may be formed during the degradation of Mg alloys. These species can be adsorbed by ceria, through the formation of stable coordination complexes, which would lead to better protection against corrosion.^32^ These cerium-stabilized species have the ability to block pathways for electrolyte adsorption, slowing down the corrosion process. Hydroxide ions released during the corrosion of the Mg alloy substrate could also be easily adsorbed on ceria surfaces.^32^ Furthermore, chloride ion ions can take up oxygen space in the CeO_2_ structure, and this association has proven to be quite stable.^32^ To sum up, the self-healing mechanism of ceria NPs may be attributed to the adsorption of chloride ions, the stabilization of Mg degradation products formed on the surface, and the physical blockage of aqueous media diffusion pathways within the coating.^32^Fig. 2 outlines the possible self-healing mechanisms of cerium-based coatings.

According to Y. Kim *et al.*^81^ and A. Pepe *et al.*,^82^ when the pH of the interface increases as Mg corrodes in the defective area, the cerium cations present in the coating exhibit a high reactivity with oxygen. The Ce^3+^ ions are hydrolyzed, and cerium hydroxide precipitates to protect the metal surface. The interaction between the Ce^3+^ and Ce^4+^ ions released from the coating and the OH^−^ ions produced by the corrosion process leads to the formation of insoluble Ce compound such as cerium oxides and hydroxides.

Surface modification of Mg alloys with CeO_2_ to improve corrosion resistance has shown promising results. For instance, Z. You *et al.*^83^ investigated the effect of cerium on the corrosion resistance of metal alloy substrates. These authors found that the insoluble cerium hydroxides (Ce(OH)_4_ and Ce(OH)_3_) precipitated on the metal surface and inhibited corrosion, thus acting as cathodic inhibitors. For their part, L. M. Calado *et al.*^84^ used cerium tri(bis (2-ethylhexyl) phosphate) (Ce(DEHP)_3_) as a pH-sensitive corrosion inhibitor capable of locally reducing the corrosive activity of Mg alloys. According to the Electrochemical Impedance Spectroscopy (EIS) results, adding 325 ppm of Ce(DEHP)_3_ enhanced the barrier properties of the coating and provided stable and long-term corrosion protection for the Mg alloy. The protective effect of Ce(DEHP)_3_ has been linked to the formation of stable Ce(OH)_3_ products as a result of an increase in local pH during the corrosion process.

Other authors^85^ have examined the effect of ceria coating on the physicochemical and corrosion resistance of the AZ31 Mg alloy. Their results show that ceria helps increase corrosion resistance by forming products with improved barrier properties that block the corrosive activity of the damaged surface. Kim *et al.*^86^ confirmed the self-healing properties of ceria-based coatings. They demonstrated that a Carboxymethyl Cellulose (CMC) coating containing hydroxyapatite and cerium ions contributes to the initial corrosion resistance of Mg alloys. Furthermore, the cerium ions stored in the CMC matrix act as active sites even after prolonged exposure to corrosive media.

Table 2 summarizes some of the results reported in the literature regarding Mg alloys coated with CeO_2_. This table also shows their corrosion potential (*E*_corr_) and corrosion density (*I*_corr_), which are key parameters to determine corrosion resistance. In summary, all ceria-based coatings deposited on different Mg alloys have proven to increase corrosion resistance, and their self-healing properties have been reported in various studies.

**Table 2:** Ceria based coatings deposited on magnesium alloys

Mg alloy	Type of coating	Coating thickness	Most relevant result	Corrosion parameters estimated *via* potentiodynamic polarization test	Biological evaluation	Ref.	Year
AZ91D	CeO_2_- and ascorbic acid (Hasc)- based coatings	∼5 μm	The incorporation of ascorbic acid into the Ce-based coating improved its anticorrosive properties. The presence of insoluble cerium precipitates and the formation of insoluble chelates are associated with improved corrosion resistance	*E*_corr_ a	Not reported	88	2018
				AZ91D: −1.501 V			
				RCe-HAsc: −0.952 V			
				*I*_corr_ b			
				AZ91D: 0.105 mA cm^−2^			
				RCe-HAsc: 5.4 × 10^−3^ mA cm^−2^			
				*I*_corr_ of the coating samples was found to be one order of magnitude lower than that of the uncoated substrate			
AZ31	CeO_2_ coating	1.1 μm and 5.3 μm	CeO_2_ coatings showed a good protective ability after immersion for 96 h in a 1 NaCl solution, which indicates their good corrosion resistance and durability	Not reported	Not reported	90	2018
AZ91D	Cerium and cerium molybdenum coating	Not reported	Cerium and cerium molybdenum coats can effectively improve the corrosion resistance of the alloy	*E*_corr_ a	Not reported	91	2018
				AZ91D: − 1.501 V			
				Ce-coat: − 1.095 V			
				Ce–Mo-coat: − 0.785 V			
				*I*_corr_ b			
				AZ91D: 0.105 mA cm^−2^			
				Ce-coat: 1.5 × 10^−2^ mA cm^−2^			
				Ce–Mo-coat: 9 × 10^−3^ mA cm^−2^			
Pure Mg	CeO_2_/ZnO coating	∼16 μm	The corrosion rate of the pure Mg drastically reduced from 10.8 to 0.81 mpy due to composite coating. During corrosion, passive layers of Ce(OH)_2_, Mg(OH)_2_, and zinc oxy-chloride were formed. The layers exhibited improved adhesion and higher corrosion resistance	*I*_corr_ b	Not reported	92	2019
				Pure mg: 1.2 × 10^−7^ mA cm^−2^			
				CeO_2_/ZnO: 9 × 10^−9^ mA cm^−2^			
				*I*_corr_ of the CeO_2_/ZnO composite coating decreased by 3 × 10^−5^ μAcm^−2^ when compared to that of pure Mg			
AZ31	Tetraethyl-orthosilicate (TEOS) and glycidoxypropyl-triethoxysilane (GPTMS) coating doped with Ce(NO_3_)_3_	Between 0.9 and 3.3 μm	Ceria in sol–gel coatings were found to deposit on the active sites of the Mg alloy providing corrosion protection	*E*_corr_ a	Not reported	85	2020
				AZ31: −1.47 V			
				TEOS + GPTMS + Ce(NO_3_)_3_: −1.46 V			
				*I*_corr_ b			
				AZ31: 6.60 × 10^−3^ mA cm^−2^			
				TEOS + GPTMS + Ce(NO_3_)_3_: 6.85 × 10^−5^ mA cm^−2^			
				The coatings synthesized with Ce(NO_3_)_3_ exhibited a reduction in *I*_corr_ of about two orders of magnitude when compared to the uncoated AZ31 alloy			
Pure Mg	Multi-layer coating of calcium, cerium, hyaluronic acid (HA), and carboxymethyl cellulose (CMC)	19.4 ± 0.09 μm	The morphology, chemical structure, and scratch tests revealed that the samples treated with Ce were the most effective in terms of self-healing and corrosion resistance. The films containing HA and CMC acted as a pathway for the diffusion of Ce ions into the film, protecting the Mg substrate and oxide film and maximizing self-healing	Not reported	*In vitro* assays using osteoblast cells showed no toxicity in ceria-based coatings. Furthermore, Ce-coated substrates exhibited good cellular expression	86	2020
					*In vivo* implantation tests in rat tibiae showed stable growth of bone marrow and osteoblasts in coatings containing ce		
LZ91	Permanganate/cerium coating	0.66 μm	Permanganate/cerium coatings were found to be more anticorrosive than chromate coatings	*E*_corr_ a	Not reported	93	2020
				LZ91: −1.64 V			
				LZ91/Permanganate/cerium coating			
				−1.53 V			
				*I*_corr_ b			
				LZ91: 1.85 × 10^−2^ mA cm^−2^			
				LZ91/Permanganate/cerium coating: 1.02 × 10^−3^ mA cm^−2^			
AZ31	CeO_2_ NPs in a hybrid epoxy–silane coating	10.4 ± 1.9 μm	Coating with 325 ppm of CeO_2_ improved corrosion protection and aided in the healing of the pitting corrosion of the AZ31 alloy	Not reported	Not reported	32	2021
WE43	Ce(DEHP)_3_ in a hybrid epoxy–silane coating	3.90 ± 0.54 μm	The barrier properties of the coating were improved. The presence of Ce(DEHP)_3_ improved the corrosion resistance. The self-healing effect of Ce(DEHP)_3_ was found to be pH-dependent	Not reported	Not reported	78	2021
AZ91	Alumina and cerium oxide sol–gel coating	Not reported	The corrosion resistance of the Mg alloy improves with increasing cerium content in the coating (up to 10% CeO_2_ content)	Not reported	No reported	94	2021
Mg-4 wt%Y	Cerium-based conversion coating (CeCC)	1.30 μm	CeCC coating retard the corrosion process of Mg alloy and decrease the corrosion rate (∼50–70% compared to bare alloy)	*E*_corr_ a	Not reported	95	2021
				Mg-4 wt%Y: −1.60 V			
				Mg-4 wt%Y –CeCC-30 s: −1.64 V			
				*I*_corr_ b			
				Mg-4 wt%Y: 5.88 10^−2^ mA cm^−2^			
				Mg-4 wt%Y–CeCC: 1.69 10^−2^ mA cm^−2^			
AZ31	Zinc–cerium, LDH/oxide	Not reported	The layered double hydroxides (LDH) were composed of the Zn^2+^ cations and the complex of the Ce^3+^ and Ce^4+^. The coating showed adequate self-healing capacity and corrosion resistance	*E*_corr_ a	Not reported	96	2021
				AZ31: −1.57 V			
				AZ31/Zn–Ce LDH/oxide: −1.26 V			
				*I*_corr_ b			
				AZ31: 1.2 × 10^−2^ mA cm^2^			
				AZ31/Zn–Ce LDH/oxide *I*_corr_: 9.8 × 10^−5^ mA cm^2^			
AZ61	CeO_2_-based composite	1.4 μm	The composite coating used is composed of the cerium conversion coating, a dense CeO_2_ layer, a porous CeO_2_ nanorods, and stearic absorbing layers. The coatings improve the corrosion resistance of Mg alloy and showed superhydrophobic properties	*E*_corr_ a	Not reported	97	2021
				AZ61: 1548.0 V			
				AZ61/Ceria-based: −1433.5 V			
				*I*_corr_ b			
				AZ61: 35.3 × 10^−2^ mA cm^−2^			
				AZ61/ceria-based: 2.0 × 10^−4^ mA cm^−2^			
AZ31	Multi-layer coating composed of MAO/phytic acid (PA)/CeO_2_	5 μm	Self-healing coatings can release cerium ions to the active sites to form a new layer and inhibit further substrate corrosion	*E*_corr_ a	Not reported	98	2022
				AZ31: −1.51 V			
				AZ31/MAO/PA/Ce: −1.62 V			
				*I*_corr_ b			
				AZ31: 7.9 × 10^−2^ mA cm^−2^			
				AZ31/MAO/PA/Ce: 1.24 × 10^−4^ mA cm^−2^			
				*I*_corr_ of the MAO/PA/CeO_2_ coating decreased by two orders of magnitude			
AZ31	Zinc–cerium LDH coating	Between 6.1 and 24.1 μm	The LDH coating composed of Zn and Ce cations on the Mg alloy has self-healing and corrosion protection properties	*E*_corr_ a	Not reported	99	2022
				AZ31: −1.57 V			
				Zn–Ce LDH-coat: −1.198 V			
				*I*_corr_ b			
				AZ31: 1.08 mA cm^−2^			
				Zn–Ce LDH-coat: 1.29 × 10^−5^ mA cm^−2^			
AZ91D	Duplex cerium-epoxy coating	Not reported	The presence of cerium in the coating provides a corrosion inhibition effect on Mg alloy	*E*_corr_ a	Not reported	100	2022
				AZ91D: −1.420 V			
				Duplex coat: −0.706 V			
				*I*_corr_ b			
				AZ91D: 0.108 mA cm^−2^			
				Duplex coat: 2.763 × 10^−6^ mA cm^−2^			
WE43C-T5	Duplex cerium-hybrid coating	2.06 μm	The addition of cerium to the coating increases the corrosion resistance of Mg alloy. Cerium exhibits self-healing properties by migrating to defect sites	*E*_corr_ a	Not reported	101	2022
				WE43C-T5: −1.66 V			
				Duplex coat: −1.59 V			
				*I*_corr_ b			
				WE43C-T5: 0.109 mA cm^−2^			
				Duplex coat: 6 × 10^−3^ mA cm^−2^			
AZ31	Calcium–cerium based LDH	22 μm	The developed coating acts as a corrosion inhibitor and exhibits self-healing properties. The release of ce, Ca and Mg ions in the corrosive medium of the Mg surface and tries to cure the defects by forming some compounds as corrosion products. This process delays the corrosion process of the metal	*E*_corr_ a	Not reported	102	2022
				AZ31: −1.61 V			
				AZ31/Ca–Ce based LDH: −1.19 V			
				*I*_corr_ b			
				AZ31: 54.72 mA cm^−2^			
				AZ31/Ca–Ce based LDH: 0.058 mA cm^−2^			

a *E*_corr_: Corrosion potential.

b *I*_corr_: Corrosion density.

In conclusion, multiple studies have been performed using ceria-based coatings deposited on Mg alloys^87^ and demonstrated their ability to reduce corrosion rates.^81,88,89^ However, little research has been done to evaluate the corrosion resistance of such coatings under physiological conditions for biomedical applications. In addition, further studies are needed to determine the parameters for obtaining biocompatible ceria coatings on Mg alloys.

## 5. Summary and discussion

Mg and its alloys are promising materials for use as implantable metals in biomedical applications. However, due to their low corrosion potential, they are susceptible to rapid degradation in aqueous solutions such as physiological environments. Rapid corrosion implies rapid dissolution, which raises concerns about how to control the rate of corrosion of Mg implants. Recent developments in the use of these biodegradable metals as implants have focused on improving their corrosion resistance. Surface modification, for instance, is one of the most efficient alternatives to address this problem. Ceria based materials have recently emerged as materials that can be used as smart coatings on Mg alloys.

Ceria NPs have been described as non-toxic materials, and their good biological responses (*e.g.*, antioxidant, anti-inflammatory, antibacterial, angiogenic, and tissue regeneration properties) have been frequently reported in recent years. Nevertheless, these particles can have a toxic effect depending on the concentration, the route of administration, and even the type of cells being analyzed.

According to the findings of various studies, CeO_2_ NPs may have selective cytotoxic effects. They can induce apoptotic processes in cancer or tumor cells, but they can also enhance the antioxidant properties of normal cells. Therefore, it is critical to recognize that the cytotoxic effect of ceria NPs depends on the type of cells used for the tests. Additionally, the effect of ceria-based materials on cell signaling pathways should be experimentally demonstrated to determine if the redox mechanisms effectively explain the selective cytotoxicity of ceria NPs or if other factors are at play.

Current biomedical research trends are analyzing the potential of CeO_2_ NPs for use in therapeutic strategies against cancer, drug delivery, antidiabetic activity, tissue regeneration, and even antibacterial applications thanks to the antioxidant properties of these materials. These studies, however, are still in the preclinical phase because the effect of these materials is still being evaluated using *in vitro* and *in vivo* models. Therefore, further research is needed to better understand the effect of these materials on the human body and their potential use in biomedical treatments. Although some problems and challenges remain unsolved, the unique physical and chemical properties of CeO_2_ NPs, as well as the significant progress made, clearly demonstrate that ceria is a fascinating, versatile, and promising material for a variety of biomedical applications.

## 6. Conclusions and trends

Current research trends in Mg and its alloys for orthopedic applications are focused on the development of smart coatings with self-healing properties that improve corrosion resistance.

Ceria-modified self-healing coatings for Mg alloys are attracting special attention in different areas of the biomedical sciences. Despite the lack of consensus on the toxicity of ceria NPs, the number of studies into their therapeutic applications has increased in recent years.

Although few studies have incorporated CeO_2_-based materials into coatings for Mg alloys, their results reveal that the corrosion resistance of Mg could be significantly improved through a self-healing process. Additionally, according to the results of *in vitro* biological evaluations, Mg coated with ceria-based materials holds promise for its potential use in biomedical applications. Even though the great biological potential of these materials is notable, their clinical application is still restricted because more studies are needed to ensure their biocompatibility and safe use in the human body.

Current trends in the field are focused on the development and use of polymers that favor the diffusion of CeO_2_ NPs onto the exposed Mg surface. Future research could concentrate on fully evaluating the biological properties of CeO_2_ NPs when implanted in bone tissue, as well as their properties when incorporated into polymeric coatings for Mg alloys.

## Conflicts of interest

The authors declare that they have no known competing financial interests or personal relationships that could have appeared to influence the work reported in this paper.

## Supplementary Material
